# Clinical Complexity of Acute Pancreatitis in a Patient With Jaundice Associated With Acute Hepatitis A, Cholelithiasis, and Recent Endoscopic Retrograde Cholangiopancreatography: A Case Report

**DOI:** 10.7759/cureus.110892

**Published:** 2026-06-15

**Authors:** Kaka Renaldi, Muhammad K Azwar, Saskia A Nursyirwan

**Affiliations:** 1 Division of Gastroenterology, Pancreatobiliary, and Digestive Endoscopy, Dr. Cipto Mangunkusumo National General Hospital, Jakarta, IDN

**Keywords:** cholelithiasis, endoscopic retrograde cholangiopancreatography, hepatitis a, jaundice, pancreatitis

## Abstract

The coexistence of gallstone disease, post-endoscopic retrograde cholangiopancreatography (ERCP) pancreatitis, and acute hepatitis A is uncommon and presents significant diagnostic and therapeutic challenges. We reported a 26-year-old man who initially presented with a three-week history of jaundice and pruritus. He was diagnosed with acute pancreatitis associated with acute hepatitis A, ERCP, and gallstones. Following initial clinical improvement, the patient’s condition deteriorated, and contrast-enhanced CT demonstrated complications in the form of infected walled-off necrosis in the lesser sac and extensive infected acute necrotic collections, without vascular complications. He underwent laparotomy with cholecystectomy, necrosectomy, and drainage. Culture-directed intravenous antibiotics were administered for 14 days alongside supportive therapy, resulting in full clinical recovery.

## Introduction

Globally, acute pancreatitis remains the most common pancreatic disease [1]. Acute pancreatitis is characterized primarily by severe abdominal pain, the risk of organ failure, and systemic inflammation, requiring appropriate and timely intervention [2]. With advancements in its management over the past decade, mortality rates have declined [3]. Identification of the underlying etiology remains of paramount importance, followed by prompt and targeted treatment. Gallstones and alcohol consumption are still the most common etiologies of acute pancreatitis. On the other hand, co-occurrence of acute pancreatitis and viral hepatitis is possible, but considered rare [4,5]. The simultaneous presence of gallstone disease, post-Endoscopic Retrograde Cholangiopancreatography (ERCP) pancreatitis, and acute hepatitis A creates substantial diagnostic and therapeutic complexity. We report such a case complicated by infected walled-off necrosis (WON) and acute necrotic collection (ANC) requiring surgical intervention. This case was reported in accordance with the 2013 CARE Case Report Guidelines [6].

## Case presentation

A 26-year-old man was referred to a tertiary care gastrointestinal endoscopy center for ERCP with a three-week history of jaundice and associated pruritus. He denied fever, abdominal pain, diarrhea, nausea, or vomiting. There was no recent travel or sick contacts. Ultrasonography demonstrated multiple cholelithiasis, and laboratory testing showed predominantly conjugated hyperbilirubinemia (Table 1).

**Table 1:** Laboratory values during admission and post-endoscopic retrograde cholangiopancreatography

Test	Result	Normal value
Admission		
Total bilirubin	4.1 mg/dL	<1.20 mg/dL
Conjugated bilirubin	2.7 mg/dL	<0.20 mg/dL
Unconjugated bilirubin	1.4 mg/dL	0.20-0.80 mg/dL
Alanine aminotransferase (ALT)	64 U/L	10-50 U/L
Aspartate aminotransferase (AST)	45 U/L	<40 U/L
Post-endoscopic retrograde cholangiopancreatography (ERCP) procedure		
Amylase	874 U/L	13-53 U/L
Lipase	2043 U/L	13-60 U/L
Leukocyte	14,870 cells/µL	4,000-10,000 cells/µL
Basophil	0.1%	0.0-2.0%
Eosinophil	0.0%	1.0-6.0%
Neutrophil	92.1%	40.0-80.0%
Lymphocyte	2.8%	20.0-40.0%
Monocyte	5.0%	2.0-10.0%
Anti-HAV IgM	Reactive	Nonreactive
HBsAg	Nonreactive	Nonreactive
Anti-HCV total	Nonreactive	Nonreactive

Liver enzyme tests demonstrated liver enzyme elevation. No viral hepatitis panel testing was done prior to referral.

ERCP was performed with selective cannulation of the common bile duct (CBD). Cholangiography demonstrated non-dilated intrahepatic bile ducts (IHBD) and common hepatic duct (CHD), with mild dilation of the CBD. A limited sphincterotomy and balloon trawl were undertaken. However, the CBD was clear, suggestive of spontaneous passage of the stone. Patient received intravenous lactated Ringer's solution and rectal nonsteroidal anti-inflammatory drug (NSAID) suppository for prophylaxis of post-ERCP pancreatitis (PEP).

Twelve hours post-procedure, he developed fever, nausea, vomiting, and epigastric pain with elevated serum amylase level and lipase, accompanied by leukocytosis and relative neutrophilia (Table 1). As this patient did not undergo multiple attempts at cannulation, other etiologies of acute pancreatitis were sought. Viral hepatitis panel tests showed reactive anti-hepatitis A virus immunoglobulin M (Anti-HAV IgM).

We concluded that the patient suffered from PEP, concurrent acute hepatitis A, and a possible bacterial infection component. He received supportive analgesics, moderate hydration with lactated Ringer's solution (1.5 ml/kg body weight/hour), as well as meropenem 1 g IV t.i.d. The patient's condition gradually improved following four days of inpatient care. Serum amylase and lipase levels subsequently dropped to 55 and 101 U/L, respectively. Alkaline phosphatase and gamma-glutamyl transferase levels were also normal.

The only remaining symptom before discharge was jaundice. We ordered additional abdominal ultrasonography for evaluation, and it showed hepatomegaly and cholelithiasis with signs of cholecystitis (Figure 1).

**Figure 1:**
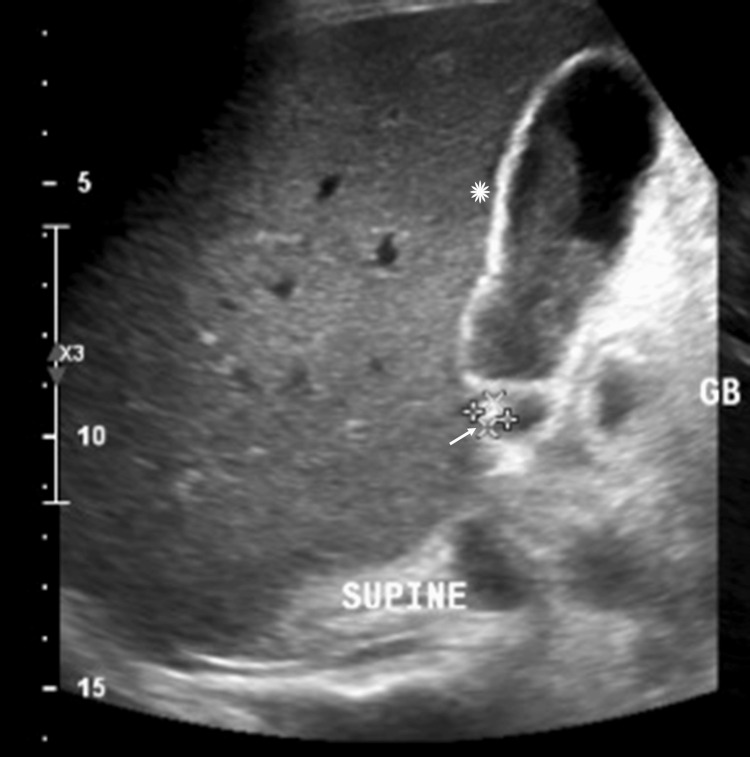
Abdominal ultrasound showing gallbladder pathologies Abdominal ultrasonography demonstrating hepatomegaly, gallbladder sludge, and a 0.6 × 0.5 cm hyperechoic lesion (white arrow) with posterior acoustic shadowing in the gallbladder neck, consistent with cholelithiasis. Gallbladder wall thickening (asterisk) is also noted.

The patient was safely discharged with improved symptoms, without urgent cholecystectomy planned during the first admission.

One day following hospital discharge, the patient started to experience abdominal pain in the right upper quadrant, accompanied by nausea. He was admitted again for further evaluation and inpatient care. CT abdomen and pelvis with intravenous contrast revealed features consistent with acute pancreatitis (Figures 2A, 2B).

**Figure 2:**
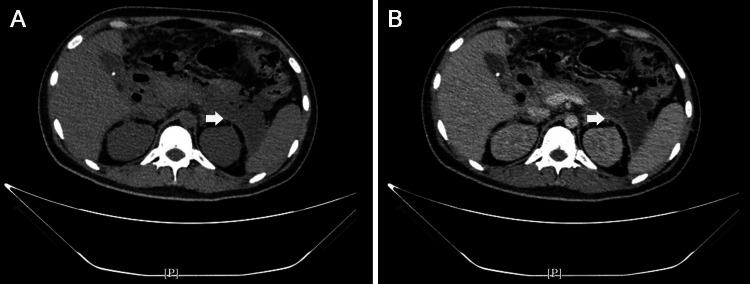
Contrast-enhanced CT of the abdomen Contrast-enhanced CT of the abdomen and pelvis showing a walled-off necrosis (white arrows). (A) Pre-contrast and (B) post-contrast images demonstrate a well-circumscribed peripancreatic collection with an enhancing wall and internal gas, consistent with infected walled-off necrosis secondary to acute necrotizing pancreatitis.

Infected WON was identified within the lesser sac. Infected ANC was present in the peripancreatic, perimesenteric, and right paracolic regions. There was no evidence of active hemorrhage, vascular complications (no pseudoaneurysm or venous thrombosis demonstrated), or biliary complications. CT Severity Index (CTSI) was 10/10, consistent with severe acute pancreatitis. In the hepatobiliary system, cholelithiasis was present without CT features of acute cholecystitis. No pleural effusion was identified.

A diagnosis of moderately severe acute pancreatitis (Modified Marshall score [7,8] was two) with infected WON and ANC, cholelithiasis, and acute hepatitis A was established. The patient was immediately referred to a digestive surgeon and underwent a laparotomy with cholecystectomy, debridement, and drainage. He received pancreatic tissue culture-directed definitive intravenous antibiotic (meropenem 1 g IV t.i.d.) and supportive care for 14 days, resulting in full clinical recovery. He was finally able to tolerate a solid diet orally and was free of abdominal pain and jaundice.

## Discussion

Acute pancreatitis is usually marked by intense abdominal pain and a systemic inflammatory response, with the potential to progress to multiorgan failure, often requiring close monitoring and aggressive medical management [2]. The global incidence of pancreatitis was largely driven by factors such as gallstone disease, alcohol consumption, smoking, and metabolic disorders, including hypertriglyceridemia. Gallstones and alcohol consumption represent the most common etiologies [2]. However, viral causes, including coxsackievirus, mumps, and hepatitis viruses, have also been described. Among these, pancreatitis associated with HAV infection is exceedingly rare, with only isolated cases reported in the literature [4,5].

The suspected etiologies of acute pancreatitis in our case include acute hepatitis A, PEP, and gallstone-related pancreatitis. In our tertiary care center where the patient’s ERCP was performed, 26.5% experienced PEP, and 52.4% were categorized as having severe pancreatitis [9]. Risk factors for PEP can be classified into patient-related and procedure-related risk factors. Definite patient-related risk factors include suspected sphincter of Oddi dysfunction, female gender, and previous pancreatitis. Meanwhile, definite procedure-related risk factors include cannulation attempts duration >10 minutes, greater than one pancreatic guidewire passage, and pancreatic injection during the procedure [10]. A previous study conducted at our center demonstrated that multiple attempts at cannulation were the only significant risk factor for PEP [9]. This patient did not experience multiple attempts at cannulation, and therefore, additional etiologies of acute pancreatitis should be sought.

Guidelines [10,11] recommend rectal NSAID (indomethacin or diclofenac 100 mg) and aggressive intravenous hydration (e.g., a 20 mL/kg bolus of intravenous Ringer's lactate, followed by an infusion rate of 3 mL/kg in the inpatient setting) for post-ERCP prophylaxis in unselected patients. Pancreatic duct stent placement was also recommended for high-risk patients. In this case, the patient had received rectal NSAIDs and intravenous fluid hydration as part of the post-ERCP prophylaxis protocol in our center. However, the patient subsequently developed PEP, illustrating that currently recommended prophylactic strategies reduce but do not eliminate the risk of this complication.

In this patient, Hepatitis B surface antigen (HBsAg) and anti-Hepatitis C Virus (HCV) antibody testing showed nonreactive results, but the anti-HAV IgM test result was reactive. Testing the viral hepatitis panel may still be relevant when managing patients with acute pancreatitis and/or cholestatic-pattern hyperbilirubinemia, as in our patient, as viral hepatitis might be linked to the development of edema of the ampulla of Vater with obstruction to the outflow of pancreatic fluid [12]. There is no evidence yet suggesting the ability of the hepatitis A virus (HAV) to reach the pancreas directly [13].

Although HAV primarily affects the liver, it may rarely give rise to extrahepatic manifestations involving the neurological, hematological, and renal systems. Pancreatic involvement is exceptionally uncommon but clinically significant, as it may complicate an otherwise self-limiting infection and create diagnostic uncertainty in the acute setting. Other proposed mechanisms of how hepatitis A causes damage to the pancreas include direct viral cytopathic effects and immune-mediated inflammatory injury [12].

Gallstones remained a possible etiology of our acute pancreatitis patient. Moreover, limited sphincterotomy was undertaken. Abdominal ultrasonography to evaluate for cholelithiasis should be performed on all acute pancreatitis cases, because gallstone-related pancreatitis is common, and it is crucial for physicians to prevent recurrent attacks [3,14]. It is recommended that patients who have undergone a biliary sphincterotomy should be referred for prophylactic cholecystectomy because the risk of recurrent disease remains high [3,15]. In our case, cholecystectomy was done during the second admission. Patients with pancreatic necrosis complicating gallstone-related pancreatitis, including our patient, require complicated decision-making between the gastroenterologist and the digestive surgery team [3]. It is important to highlight that patients with acute biliary pancreatitis are recommended to undergo cholecystectomy early, preferably before discharge [3]. Same-admission cholecystectomy was shown to reduce healthcare expenditure [3,16]. Delayed cholecystectomy might result in a longer length of stay [3,17].

In this case, local complications were identified, namely, infected WON and infected ANC. Pancreatic necrosis is well recognized to be associated with prolonged hospitalization. Careful monitoring for resolution or progression of organ failure(s) is imperative, as the risk of organ failure appears comparable between infected and sterile necrosis.

Ultimately, this case highlighted the difficulty of determining the etiology of jaundice and pancreatitis in patients with atypical clinical features. Initially, the patient was referred to our hospital with jaundice presumed to be due to choledocholithiasis based on the abdominal ultrasound showing multiple gallstones and conjugated hyperbilirubinemia, with only slight elevation of liver enzymes. However, viral hepatitis testing had not yet been performed, and neither magnetic resonance cholangiopancreatography (MRCP) nor endoscopic ultrasound (EUS) was obtained before ERCP.

In retrospect, there is a possibility that acute hepatitis A may have contributed substantially to the patient's initial cholestatic presentation. However, the mild transaminase elevation and predominantly conjugated hyperbilirubinemia at presentation were not typical of classic acute hepatitis A. Therefore, the diagnosis remained challenging, and choledocholithiasis, viral hepatitis, and gallstone-related disease were all considered potential contributors to the clinical presentation.

## Conclusions

Proper identification of the etiology of acute pancreatitis remains of paramount importance, followed by prompt and appropriate management. Our patient developed acute pancreatitis, which may have been associated with acute hepatitis A, ERCP, and cholelithiasis. The coexistence of acute hepatitis A infection, gallstone disease, and subsequent ERCP created significant diagnostic uncertainty regarding the primary etiology of both the initial jaundice and the pancreatitis. Contrast-enhanced CT demonstrated complications in the form of infected WON in the lesser sac and extensive infected ANC, without vascular complications. Following laparotomy with cholecystectomy, necrosectomy, and drainage, together with supportive therapy and culture-directed intravenous antibiotics, the patient achieved full clinical recovery. This case highlights the importance of maintaining a broad differential diagnosis in patients presenting with jaundice and pancreatitis. Alternative etiologies should be carefully considered before invasive biliary interventions when clinically feasible. Current evidence supports the benefit of early cholecystectomy, preferably before discharge, in the management of acute biliary pancreatitis.
